# The experience of nurses to reduce implicit rationing of nursing care: a phenomenological study

**DOI:** 10.1186/s12912-023-01334-5

**Published:** 2023-05-19

**Authors:** Hui Qin Li, Peng Xie, Xia Huang, Shan Xia Luo

**Affiliations:** 1 Mental Health Center, West China School of Nursing, West China Hospital, Sichuan University, No. 37 Guoxue Road, Chengdu, Sichuan Province 610041 P.R. China; 2 Surgical Anesthesia Center, West China School of Nursing, West China Hospital, Sichuan University, No. 28 Telecom South Street, Chengdu, Sichuan Province 610041 P.R. China

**Keywords:** Implicit rationing of nursing care, Nursing management, Nurses, Qualitative research, Phenomenological study

## Abstract

**Background:**

Implicit rationing of nursing care can adversely affect patient safety and the quality of care, and increase nurses’ burnout and turnover tendency. Implicit rationing care occurs at the nurse-to-patient level (micro-level), and nurses are direct participants. Therefore, the strategies based on experience of nurses to reduce implicit rationing care have more reference value and promotion significance. The aim of the study is to explore the experience of nurses to reduce implicit rationing care, thereby to provide references for conducting randomized controlled trials to reduce implicit rationing care.

**Methods:**

This is a descriptive phenomenological study. Purpose sampling was conducted nationwide. There are 17 nurses were selected and semi-structured in-depth interviews were conducted. The interviews were recorded, transcribed verbatim and analyzed via thematic analysis.

**Results:**

Our study found that nurses’ reported experience of coping with implicit rationing of nursing care contained three aspects: personal, resource, and managerial. Three themes were extracted from the results of the study: (1) improving personal literacy; (2) supplying and optimizing resources and (3) standardizing management mode. The improvement of nurses’ own qualities are the prerequisites, the supply and optimization of resources is an effective strategy, and clear scope of work has attracted the attention of nurses.

**Conclusion:**

The experience of dealing with implicit nursing rationing includes many aspects. Nursing managers should be grounded in nurses’ perspectives when developing strategies to reduce implicit rationing of nursing care. Promoting the improvement of nurses’ skills, improving staffing level and optimizing scheduling mode are promising measures to reduce hidden nursing rationing.

## Introduction

Implicit rationing of nursing care is defined as the refusal or failure to implement necessary nursing measures for patients due to lack of nursing resources (staffing, skill set, time) [1], which includes unfinished care, missed care and care left undone, [2] and has been a topic of increasing interest for nursing researchers all over the world [3–5].

With the increasing diversity of people’s health needs and major changes in the complexity of the treatment and nursing process, the health care system has continuously increased requirements for the number of nurses and the quality of nursing care [6, 7]. However, medical systems all over the world are facing the problem of a shortage of nurses [8], so implicit rationing of nursing care is often reported [2, 9–11]. Implicit rationing care can adversely affect patient safety and the quality of care [4, 12]. A high level of implicit rationing care increases the incidence of pneumonia, falls, pressure injuries and blood infections in hospitalized patients, and increases the 30-day mortality rate of hospitalized patients [12, 13]. In addition, failing to complete all necessary care can make nurses feel guilty, which in turn increases nurses’ burnout and turnover tendency [13, 14]. Therefore, it is vital to take measures to reduce implicit rationing care.

Only one intervention study to date has verified the effectiveness of teamwork in reducing implicit rationing care [15], and no other randomized controlled trials have explored strategies to reduce implicit rationing care. Although there are many studies on implicit rationing of nursing care, these studies have the following problems: (1) Attention has been paid to the influencing factors of implicit rationing of nursing care[16–19], such as working environment, leader style, team atmosphere and fatigue, but the experience of nurses on implicit rationing of nursing care has not been explored; and (2) Most studies are quantitative studies[20–23], such as cross-sectional surveys, and lack qualitative research data. Implicit rationing care occurs at the nurse-to-patient level (micro-level), and nurses are direct participants [24]. Some scholars have proposed that strategies should be developed based on nurses’ views to improve nursing services and nursing quality [25]. In qualitative research, the researcher can understand the perspective of the respondent from the micro level and the perspective of the respondent by going deep into the subjective world of the respondent. To date, only one qualitative study has explored nurses’ views on recessive care rationing, but this study was from developed countries and focused on emergency department/hospital nurses, lacking research results from developing countries, and did not explore the perspectives of nurses in other departments [26].

The research question of this study is: What is the experience of nurses in developing countries in reducing implicit rationing of nursing care? Descriptive phenomenology can present individual subjective experience, describe the real situation of the phenomenon, and stimulate individual experience and feelings with more breadth and depth [27]. Therefore, to answer this question, a descriptive phenomenological research was conducted to explore the experience of nurses to reduce implicit rationing care, so as to provide references for conducting randomized controlled trials to reduce implicit rationing care.

## Methods

### Aim

The aim of the study is to explore the experience of nurses to reduce implicit rationing care, thereby to provide references for conducting randomized controlled trials to reduce implicit rationing care.

### Design

We conducted a qualitative study using descriptive phenomenology, also known as eidetic phenomenology, created by Husserl and colleagues as a methodological framework for qualitative health research [28].

### Participants

Nurses are participants in our study. Inclusion criteria: (1) on-the-job nurses working in hospitals; (2) participating in the direct care of patients. Exclusion criteria: (1) not on duty during the study period (such as asking for leave, vacation or going out to study); (2) refresher nurses and trainee nurses; (3) nurses unwilling to participate in our study. In order to ensure sufficient heterogeneity of the sample and obtain more extensive information, purpose sampling was carried out based on the level and location of the hospitals to which the respondents belong, as well as the department, seniority, education, marital status, gender of the respondents.

### Data collection

One-to-one semi-structured interviews underpinned by phenomenological methods (bracketing, intuiting, analysing and describing) were used for data collection in this study [29]. Before the interviews, the domestic and foreign literatures related to the limitation of nursing service was searched to understand the research status and the known content of the implicit rationing of nursing care, and then combined with the qualitative interview outline design skills to preliminarily establish the interview outline framework. Three nurses who met the inclusion criteria and exclusion criteria were pre-interviewed, and preliminary outlines were discussed among the research teams. Based on the pre-interview feedback and discussion results, the final interview outline was determined.

All interviews were conducted at locations selected by the interviewees, and nobody present besides the participants and researchers, which can ensure the ease and harmony of the interview scenarios and maximize the exploration of the subject content. The experienced female interviewer used communication skills such as clarification, repetition, retelling, reflection, and summary, and recorded the interview process to ensure the accuracy and comprehensiveness of the interview content. During each interview, the interviewer observes the interviewee’s tone, expression and actions and other non-verbal expressions, and records them.

At the end of each interview, the interviewer makes a brief summary of the interview content and narrates it to the interviewee. If there is any deviation, the interviewee can correct and supplement it to ensure the accuracy of information acquisition. Furthermore, the sociodemographic characteristics of the interviewees were investigated, including gender, age, seniority, marital status, job title, and department, to explore the views of nurses with different sociodemographic characteristics.

### Data analysis

The recordings obtained from the interviews were transcribed verbatim by an administrative collaborator with considerable transcription experience, and the tone and pauses of the interviewees were recorded when transcribed, and the recordings and transcripts were checked repeatedly to ensure the accuracy of the original data. Each transcript was read by XP or LHQ to allow emerging insights to be incorporated into the ongoing data collection [30, 31]. Colaizzi’s seven-step data analysis method in phenomenological research was used in this study, which focuses on identifying the common nature of all participants’ experiences, and data are derived from researchers’ observations and interviews [32, 33]. The transcripts were imported into Nvivo 12, and then use thematic analysis (TA) to analyze and generate the initial code. Afterwards, relocated these codes and combined these codes to consider duplication, thus forming an organizational theme and a global theme. A co-author (Peng Xie) reviewed the interview records and topics to ensure they were based on the original data. Finally, the interviewees were invited to verify the themes to provide feedback from the participants on the researcher’s interpretation of their answer.

### Validity and rigour

The respondents were interviewed by an experienced researcher and all interviews were recorded. The recordings obtained from the interviews were transcribed verbatim, and the tone and pauses of the interviewees were recorded when transcribed, and the recordings and transcribed texts were checked repeatedly to ensure the accuracy of the original data.

## Results

### Sociodemographic characteristics of respondents

The data reached saturation after 17 nurses were interviewed, with each interview duration ranging from 29 to 67 min. Among the 17 interviewees, 7 (41.18%) were men and 10 (58.82%) were women. There are 6 respondents in the age range of 30–39. Respondents with 5–10 years of work experience accounted for 47.06%. The highest level of education of most of the respondents (58.82%) was university degree. The vast majority (76.47%) of respondents are married. As far as the department where the respondent is located, the distribution of the respondent in each department is relatively even. Detailed sociodemographic characteristics are shown in Table 1.

**Table 1:** Sociodemographic characteristics of respondents

Characteristics	Respondents	
	N	%
Gender		
Male	7	41.18
Female	10	58.82
Age, year		
18–29	4	23.53
30–39	6	35.29
40–49	4	23.53
50–59	3	17.65
Seniority		
＜5	5	29.41
5–10	8	47.06
≥ 10	4	23.53
Marital status		
Married	13	76.47
Unmarried	4	23.53
Highest level of education		
Associate degree	3	17.65
University degree	10	58.82
Master degree or/and above	4	23.53
Job title		
Nurse	11	64.71
Nurse management	6	35.29
Department		
General inpatient department	5	29.41
Outpatient Department	3	17.65
Emergency department	5	29.41
Intensive care unit	4	23.53

### Themes

**Fig. 1:**
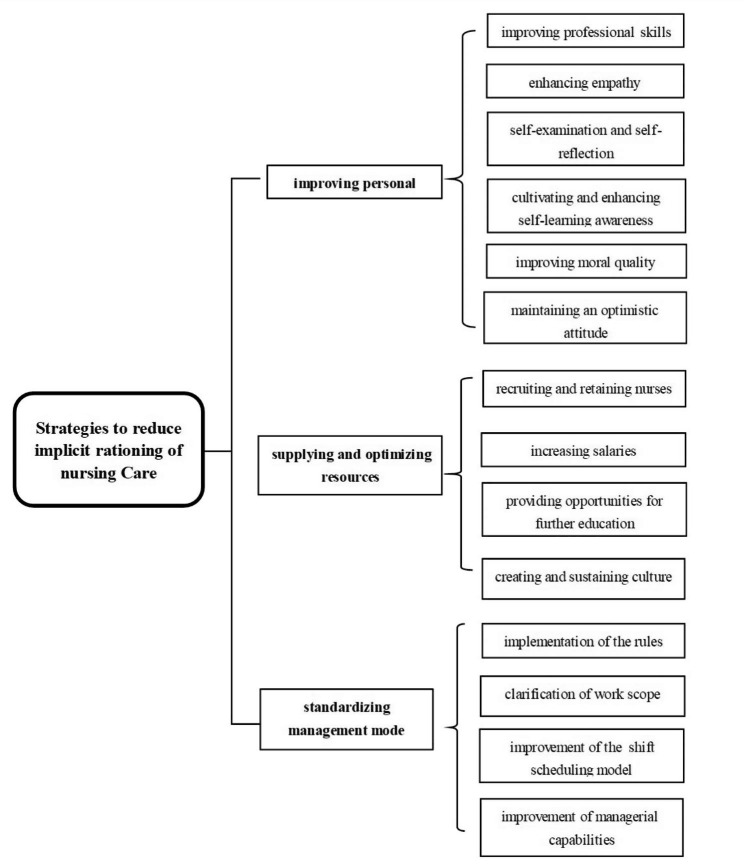
Themes Framework

Three themes have been formed, namely “improving personal literacy”, “supplying and optimizing resources” and “standardizing management mode”. Figure 1 shows the themes framework.

#### Improving personal literacy

This theme includes six sub-themes, which are improving professional skills, enhancing empathy, self-examination and self-reflection, cultivating and enhancing self-learning awareness, improving moral quality, and maintaining an optimistic attitude.

#### Improving professional skills

Adept professional skills are the guarantee of work efficiency and quality, so professional skills should be improved to promote work efficiency. The same thing can be done in a much shorter time by a nurse with adept skills, which frees up time to meet other needs of patients (N11).

#### Enhancing empathy

Empathy is the prerequisite for understanding the true feelings of patients. Many nurses reported that they should strengthen their empathy, know how to think from the perspective of patients, and learn to sympathize and understand patients, which is conducive to reducing implicit rationing of nursing care. Nursing is a career with warmth. Thinking about problems from the perspective of patients and understanding their real needs is conducive to improving the quality of nursing care (N17). In addition, strong empathy makes it possible to prevent the neglect of psychological support and health education for patients in nursing work (N3).

*“I think we should improve our empathy. Nursing is a career with warmth. If you think more from the perspective of patients, maybe we will do better“*(N4).

#### Self-examination and self-reflection

Nurses have the most contact with patients and can find the changes of patients’ condition earlier. Always keep in mind that nursing is not a mechanized operation. Nurses must have their own ideas and actively conduct self-examination in their work, so as to discover deficiencies in time and correct them (N9).

*“Self-inspection is really needed in the work…Check whether the work of this shift has been completed and done well. Otherwise, it is easy to miss the work, which will bring additional work burden to the colleagues of the next shift“(*N14).

#### Cultivating and enhancing self-learning awareness

Many technologies and concepts are developing rapidly. Continuous learning is conducive to understanding and mastering new technologies and concepts, so as to better serve patients (N12). Therefore, cultivating and improving the awareness of autonomous learning and constantly learning new technologies and knowledge can promote the improvement of nurses’ knowledge reserve and reduce the implicit rationing of nursing care at work.

*“Economy and technology are developing, if you do not learn, you will not know a lot of things, and do not know how to do well, …The awareness of autonomous learning is very important. It is the driving force for everyone to continue learning” (N8).*

#### Improving moral quality

Implicit rationing of nursing care is influenced by the moral character of the staff. Generally speaking, the level of moral character is positively correlated with the level of conscientiousness and nurses with high level of moral character will do things well seriously even without the supervision of others(N6). Improving the morality of nurses may reduce the occurrence of implicit rationing of nursing care.

*“If nurses have no sense of morality, they may ignore some of the care they think has little effect on the prognosis of patients, but the care that nurses should do, so I think the morality of nurses should be improved“*(N5).

#### Maintaining an optimistic attitude

Mentality is very important to the effectiveness of work. A pessimistic person will ignore the two sides of things and only think of the negative side of things, which will make the nurse lose enthusiasm for work (N17). Keeping an optimistic attitude can enable staff to carry out their work without losing motivation due to too many negative views, and promote the efficiency and quality of work.

*“A pessimistic attitude has a negative impact on our work, … and maintaining an optimistic attitude will make our positive, so as to ensure the completion of the work“(N2).*

#### Supplying and optimizing resources

The theme includes four sub-themes, which are recruiting and retaining nurses, increasing salaries, providing opportunities for further education, and creating and sustaining culture.

#### Recruiting and retaining nurses

The root cause of implicit rationing of nursing care is the imbalance between medical resources and health needs, especially the shortage of nurses. In the case of the shortage of nurses, nurses need to undertake a large enough task, but the development of high-quality nursing has put forward higher requirements for nurses, and further increase the workload of nurses, which increases the turnover rate of nurses, and then cause a new round of bad cycle. Therefore, the most basic strategy to reduce the implicit rationing of nursing care is to recruit new nurses and reduce the nurse turnover rate to tackle the shortage of nurses.

*“Although we are recruiting new nurses every year, our manpower is simply not enough. The separation rate of nurses every year is also extremely high … It is important to recruit new nurses, but we should also find ways to keep the working nurses“(N15).*

#### Increasing salaries

It is the common expectation of nurses that pay and gain are proportional to each other. At present, the workload of nurses is very large, but the salary is very low, which has a negative impact on nurses’ investment in work (N7). Increasing salary can mobilize nurses’ work enthusiasm, which may be an effective means to reduce the occurrence of limited nursing services.

*“I do a lot of things every day, but the salary is still very low, so my motivation is not high“(*N10).

#### Providing opportunities for further education

It is very necessary to learn from each other. However, hospitals have their own limitations. Perhaps the equipment and concepts of some hospitals are not advanced enough, so the staff from them need more opportunities to enter advanced hospitals for further training(N13). That helps nurses broaden their horizons and increase their knowledge, so that nurses can respond more calmly at work.

*“Like a frog at the bottom of a well, if you don’t understand advanced things, it will affect the quality of your work … It is very necessary to provide opportunities to study in more advanced hospitals … Only after you have a comprehensive understanding of equipment, diseases, and concepts can you do a better job”*(N7).

#### Creating and sustaining culture

Positive culture can affect people’s thoughts, and then affect people’s behavior and habits for a long time(N1). It is necessary to create and sustain a permanent hospital culture to improve the spiritual beliefs of the staff, and the cultural construction should aim to improve the nurses’ ethical awareness and awareness of teamwork, which may be a long-term effective way to reduce the implicit rationing of nursing care.

*“Establish a permanent hospital culture, especially nursing culture and spiritual belief culture, to improve people’s spiritual level … The most important thing is spiritual belief and cultural construction“(N2).*

#### Standardizing management mode

The theme includes four sub-themes, which are establishment and implementation of the rules, clarification of work scope, improvement of the scheduling model, and improvement of managerial capabilities.

#### Establishment and implementation of the rules

Strict rules and regulations can not only regulate the staff’s code of conduct, but also form a unified judgment standard. Establishing rules to specify what must be done and clear punishment will make nurses not allow imperfections in their work. Some nurses also put forward that the strict implementation of the rules is a powerful guarantee to promote nurses to improve their work and reduce implicit rationing of nursing care (N7, N13).

*“Rules must be established to stipulate what cannot be ignored and must be completed, so that nurses will not allow imperfections in their work” (N12).*

#### Clarification of work scope

Many nurses proposed that the scope of work of nurses should be clarified and priority should be given to completing nursing related tasks. Nursing care is the last link of the relationship between hospital management and patients, and the content to be completed is very tedious. The concept of high-quality nursing makes nurses often complete tasks that are not related to nursing, such as logistics, preparation of materials, bureaucratic and administrative tasks(N4). Also, due to the impact of the covid-19 pandemic, some nurses act as temporary security guards(N17). Therefore, Clarifying the work scope of nurses may be a promising way to reduce the implicit rationing of nursing care.

*“We do not have a secretary, so many administrative tasks and material preparation are done by nurses. Sometimes nursing-related things are not done, but some administrative tasks must be completed. . . The responsibilities of nurses and the scope of our work should be clarified” (N6).*

#### Improvement of shift scheduling model

Shift has an impact on nursing work. Some nurses believe that the time of 12 h shift is more flexible than 8 h shift and they can better organize their work, that is, it is easier to make priority arrangements and complete unfinished nursing care (N3, N14). The improvement of the scheduling model is mainly reflected in the shifts and personnel allocation of each shift. In addition to the change in the length of each shift, nurses expect fixed partners in each shift. They believe that working with fixed partners is conducive to achieving good teamwork, promoting the improvement of work efficiency, so as to reduce implicit rationing of nursing care (N16, N17).

*“Changing the working hours of each shift to 12 h will be more conducive to work” (N8).*

#### Improvement of managerial capabilities

The improvement of managers’ ability is very important to reduce implicit rationing of nursing care. Capable leaders can be trusted and admired by nurses. Nurses will regard them as role models to learn from and demand themselves according to their standards of behavior (N1, N9). In addition, when nurses’ work is insufficient, competent managers will provide effective demonstration and guidance to nurses, which is conducive to the reduction of implicit rationing of nursing care.

## Discussion

The aim of the study is to explore the experience of nurses to reduce implicit rationing of nursing care. To the best of our knowledge, our study is the first qualitative study to explore experience to reduce implicit rationing of nursing care from the perspective of nurses. Three themes have been identified: in our study, which is “improving personal literacy”, “supplying and optimizing resources” and “standardizing management mode”. Furthermore, corresponding sub-themes have been determined for each theme. The results of this study can provide references for nursing managers to formulate strategies to reduce the implicit rationing of nursing care and provide references for other scholars to carry out randomized controlled trials to reduce implicit rationing of nursing care.

In semi-structured interviews with nurses, it is found that the improvement of nurses’ own qualities such as professional skills, empathy, and morality are the prerequisites for reducing implicit rationing of nursing care. High-quality clinical nursing practice needs to be guaranteed by the excellent professional skills of nurses, and consistent moral reasoning based on their own moral principles can effectively guide professional performance [34, 35]. Moreover, moral sensitivity is the ability to identify ethical issues, and a lack of it may impair the quality of clinical practice [36, 37]. Moral sensitivity in nursing lies in understanding the vulnerability of the patient [38]. Some scholars believe that the code of ethics in daily clinical practice should be conveyed to nurses, so that they can have a deep understanding, which will help ensure the quality of health care [39, 40]. Furthermore, empathy is of great value in humanized nursing [41–44]. It can improve clinical practice and promote teamwork and patient-centered care [45–47]. In summary, it is necessary to carry out moral education and empathy education for nurses in clinical practice to promote the improvement of their moral and empathy levels, thereby reducing the implicit rationing of nursing care.

Keeping an optimistic mentality is one of the strategies that many nurses believe is conducive to reducing the implicit rationing of nursing care. Luthans’ research showed that nurses with an optimistic mentality are more engaged and more efficient at work, and when faced with problems, medical staff with an optimistic mentality will not escape, but will seek effective solutions to the problems [48]. In addition, optimistic employees are more likely to seek support from others and show more trust in colleagues and organizations, thereby providing greater effectiveness in the care provided to patients [49].

In this study, nurses believe that the supply and optimization of resources is an effective strategy to reduce the implicit rationing of nursing care, which is reflected in human resources, economic resources, cultural resources, and learning resources. Lower nurse staffing levels are closely associated with higher levels of implicit rationing of nursing care [50]. The shortage of nurses is a huge global challenge. With the increase of population aging and chronic disease burden, and the change of nursing concept, the demand for the number and service quality of nurses continues to increase, which further aggravation the shortage of nurses. In addition, nurses often complete tasks that are not related to nursing, such as logistics, preparation of materials, bureaucratic and administrative tasks, which further increased the workload of nurses. The imbalance between nurse shortages and heavy workloads exacerbates the implicit rationing of nursing care. The education background of nurses is an important factor related to the limitation of nursing services [51]. Therefore, the recruitment and retention of nurses, especially highly educated nurses, may be fundamental to improving the number of nurse staffing and reducing the limitations of nursing services. Satisfying nurses’ job preferences may be an effective way to recruit and retain nurses. The results of many previous studies have shown that salary is critical to nurse recruitment and retention [52–54]. In addition, the nurses interviewed in this study expect to get reasonable compensation for their efforts. Therefore, it is necessary to recruit new nurses and improve the salary and welfare of nurses to reduce the separation rate of nurses. On the other hand, it is necessary to clarify the scope of work of nurses and pay attention to the rationality of manpower allocation. It may be an effective way to engage more experienced nurses with high working years in nursing related work, and to engage nurses with medium and low working years in cooperation.

The construction of culture and more opportunities for further study have been affirmed by nurses. Nursing culture may influence nurses’ choice of working methods during busy periods [55]. Organizational culture affects nurses’ job satisfaction [56]. Studies have shown that when a hospital has a high-level organizational culture, nurses are more motivated to work [57]. The study confirmed that activities to support and respond to nurses’ needs are conducive to improving job satisfaction and reducing implicit rationing of nursing care [58]. Therefore, for countries and regions with poor economy, establishing their own long-term cultural system and meeting the needs of nurses’ further study may be the focus of the strategy to reduce the implicit rationing of nursing care.

The clear scope of work has attracted the attention of nurses. Due to the passive position of nurses in the health care system, nurses also report the implementation of non-nursing tasks in reality. Non-nursing tasks are defined as nurses performing all tasks outside the scope of their practice, including tasks within their duties and tasks outside their duties. A research reported that nurses perform non-nursing tasks at a higher rate in developing countries [59]. The performance of non-nursing tasks increases the risk of missed nursing care [60]. However, in essence, nurses seem to face the risk of performing functions outside their responsibilities. From the perspective of organization, they need to coordinate the work of all aspects. From the perspective of professional ethics, they need this flexibility in order to better take care of patients [61]. However, in the situation of shortage of nurses in world, nurses’ energy and time can not play an important part in patients care when performing tasks lower than nurses’ skills. Therefore, this situation needs to be changed fundamentally, the scope of nurses’ work should be clarified, and the number of non-nursing tasks performed by nurses should be reduced. In terms of policy, the country should pay attention to the importance and value of nurses, so that nursing profession can have more say in health care.

The improvement of shift scheduling model is an effective strategy for nurses to reduce the implicit rationing of nursing care. In terms of shifts, nurses believe that compared with 8-hour shifts, 12-hour shifts allow them have more time to ensure the completion of nursing work. Saville’s study also proved that the incidence of nursing service restriction will be reduced under 12 h shift scheduling [62]. In addition to the length of work, the fixed combination of staff has also attracted the attention of nurses. Many studies have shown that when nurses are in short supply, a relatively fixed grouping in nursing management is conducive to more efficient completion of work and effective teamwork [63–65]. Effective teamwork plays an important role in maintaining the continuity of nursing and reducing the implicit rationing of nursing care, which should be paid attention to by leaders and nurses [51]. Moreover, one study confirmed that fixed work arrangements are associated with likelihood of less overtime and shift change [66]. The continuity of nursing care is crucial. For example, the same nurse cares for the same patients for several consecutive days, which can promote nurses’ understanding of the needs of patients and improve the quality of care. In summary, nursing managers should improve the shift method and fix the staff in each shift (such as implementing 12 h shift scheduling), and emphasize the rationality of manpower allocation and the continuity of nursing, so as to reduce the occurrence of implicit rationing of nursing care.

It should be noted that the improvement of managers’ abilities (such as executive ability, leadership.) is an effective strategy to reduce implicit rationing of nursing care. A study has shown that the assistance and support of managers in the workplace can touch the consciousness fluctuation of nurses, and then affect the behavior of nurses [67]. As the nurses in our study said, competent managers are more likely to give more guidance and support, which will improve the work enthusiasm of nurses. Previous studies have shown that the ability and leadership of managers have an important impact on implicit rationing of nursing care [68–70]. Therefore, it is necessary for managers to improve their abilities in various aspects through multiple ways, so as to reduce the occurrence implicit rationing of nursing care.

### Strengths and limitations

Like other qualitative studies, this study was purposefully sampled according to the socio-demographic characteristics of the respondents, which ensured the heterogeneity of the sample. In addition, this study did not restrict the departments in which the nurses worked, which obtained broader information on reducing implicit rationing of nursing care. This study also has some limitations. Although interviewees have been informed that the interview contents are confidential, due to the sensitivity of the topic about implicit rationing of nursing care, some nurses may conceal something in the interview, which may make the results biased to a certain extent.

## Conclusion

This study explored what nurses consider to be effective strategies for reducing implicit rationing of nursing care. The study found that nurses believe that the strategies to effectively reduce the implicit rationing of nursing care are multifaceted, which are reflected in three themes: “improving personal quality”, “providing and optimizing resources” and “standardizing management mode”. Nursing managers should be grounded in nurses’ perspectives when developing strategies to reduce implicit rationing of nursing care, so that the strategies can be more effective. Our study enriched relevant articles on implicit rationing of nursing care, and provided more accurate suggestions and references for future intervention plans to reduce implicit rationing of nursing care and improve nursing quality.

## Data Availability

The datasets used and/or analysed during the current study are available from the corresponding author on reasonable request.
